# Learning from community voices about lateral violence and lateral empowerment: a scoping review of grey literature

**DOI:** 10.1080/00049530.2024.2353055

**Published:** 2024-05-27

**Authors:** Yvonne Clark, Anneka Bowman, Rachel Elovaris, Treena Clark, Naomi Thornthwaite, Karen Peterson, Karen Hawke, Feda Ali

**Affiliations:** a Aboriginal Communities and Families Health Research Alliance (ACRA), SAHMRI, Adelaide, Australia; b Faculty of Psychology, Justice and Society, University of South Australia, Adelaide, Australia; c Department of Paediatric and Reproductive Health, University of Adelaide, Adelaide, Australia; d Stillbirth Centre for Research Excellence, Mater Research, University of Queensland, Queensland, Australia; e Faculty of Design, Architecture and Building (DAB), University of Technology, Sydney, Australia; f Department of Public Health, Flinders University, Adelaide, Australia

**Keywords:** Aboriginal, Torres Strait Islander, lateral violence, lateral empowerment, child, youth, adolescent, Australia

## Abstract

**Background:**

Aboriginal and Torres Strait Islander youth are vulnerable to racism, trauma and Lateral Violence (LV) where negative feelings and behaviours are directed towards members within their own oppressed group. Conversely, Lateral Empowerment (LE) is the collective prevention and repair of the effects of LV and promotes resilience and strength. There is limited peer reviewed literature directly relating to LV and LE.

**Objective:**

This review focuses on grey literature to gain greater insight into the understanding and experiences of LV/LE among Aboriginal and Torres Strait Islander young people within Australia.

**Method:**

This grey literature scoping review identified N = 38 documents between January 1980 and September 2023 related to LV or LE to gain a greater insight into the understanding of LV and LE among the Aboriginal and Torres Strait Islander community outside of published publications.

**Results:**

The results elucidated that the experience of LV for Aboriginal and Torres Strait Islander youth is largely based upon internalised racism pertaining to Aboriginal identity. Strength-based gender-specific approaches which focus upon positive cultural experiences, the use of lived experience mentors and the inclusion of family were identified as the foundation for LE.

**Conclusion:**

The grey literature review highlights that the Aboriginal and Torres Strait Islander community are taking an active role in addressing and preventing LV through culturally informed practices and approaches based upon the truth telling of Australia’s colonial history. There remains the need for specific approaches directed at Aboriginal and Torres Strait Islander youth to help prevent and address LV/LE.

## KEY POINTS

**What is already known about this topic**:

1. LV is an emerging area of discourse within Aboriginal and Torres Strait Islander Communities.
2. LV is prevalent within Aboriginal and Torres Strait Islander communities as a symptom of the ongoing oppression of colonisation, with young people particularly vulnerable and most at risk.
3. LE is a relatively new term that aims to counteract LV by empowering Aboriginal and Torres Strait Islander people to have control over their lives.

**What the topic adds**:

1. This analysis provides a thorough review of unpublished information (“grey literature”) pertaining to LV and LE for Aboriginal and Torres Strait Islander people within Australia, with a specific focus on young people.
2. Insight into the voices of Aboriginal and Torres Strait Islander young people and their communities in relation to their understanding and lived experiences of LV/LE.
3. Insight into the experiences of Aboriginal and Torres Strait Islander youth and their communities with regard to approaches to counteract the effects of LV.

## Introduction

### Lateral violence (LV)

Contemporary definitions of lateral violence (LV) describe it as the way people from oppressed groups direct their rage, fear, shame, anger and dissatisfaction towards themselves and other members of their own community due to their feelings of oppression and the continued experience as part of settler-colonialism (Australian Human Rights Commission, 2011c; Clark et al., 2016; Whyman et al., 2023). The conceptualisation of LV originally emerged from notable writings pertaining to colonialism and slavery literature (Fanon, 1967; Freire, 1970). LV can encompass feelings of distrust, mistrust and jealousy with behaviours such as gossiping, bullying, shaming, social exclusion and various forms of violence including domestic and family violence (DV and FV, respectively), social, physical, psychological, economic and spiritual violence (Clark et al., 2016).

Oppression theory describes how the lasting impacts of colonisation underpin the creation of LV among Aboriginal and Torres Strait Islander peoples in Australia (Vaditya, 2018). Wishing to avoid social disadvantage and ostracisation resulting from racist policies, the oppressed group attempts to follow the dominant group’s social norms, rules and laws. This assimilation process can create a collective sense of self-loathing and low self-esteem, resulting in strong behavioural undercurrents known as internalised oppression (Dudgeon et al., 2000). The oppressed group can develop deeply rooted resentment or anger towards the dominant group yet is unable to act against the dominant group, and resentment is turned inward towards the self or members of their own group enacted as LV. As described by Frankland and Lewis and quoted in the 2011 Social Justice Report, “When we are consistently oppressed, we live with great fear and anger, and we often turn on those who are closest to us” (Australian Human Rights Commission, 2011c, p. 52).

### Impact of lateral violence on aboriginal and Torres Strait Islander youth

Evidence from a variety of sources suggests that Aboriginal and Torres Strait Islander children and young people are particularly vulnerable to LV (G. Charles & DeGagné, 2013; Clark, 2017; Coffin et al., 2010; Herrenkohl et al., 2022). Marcia Langton (2008) argued:

Those most at risk of LV in its raw physical form are family members and, in the main, the most vulnerable members of the family: old people, women and children. Especially the children. (p. 50)

Racism as a form of oppression has a considerable influence on social and emotional wellbeing (SEWB) outcomes for young people. Witnessing and experiencing racism and LV can be internalised and normalised at a very young age, leaving children and adolescents at risk emotionally, mentally, spiritually and physically (Calma & Priday, 2011; Clark & Augoustinos, 2015; Priday et al., 2011; Svetaz et al., 2020). A systematic review by Priest et al. found a strong and consistent relationship between racial discrimination and negative mental health outcomes for young people, this means that young people could be prone to disorders such as anxiety, depression and psychological distress (Priest et al., 2013). A longitudinal Australian study on Aboriginal and Torres Strait Islander children aged 5–10 years old found an increased risk of asthma and obesity for children experiencing direct and prolonged racism (Shepherd et al., 2017). Collectively, racism, oppression and the effects of intergenerational trauma have led to young Aboriginal and Torres Strait Islander people experiencing LV, with dire consequences (Priday et al., 2011). The vulnerability of Aboriginal and Torres Strait Islander youth is evident in the epidemic levels of suicide among this group. Rates of suicide are three times higher in Aboriginal and Torres Strait Islander youth compared to non-Aboriginal and Torres Strait Islander youth, and suicide is the leading cause of death among Aboriginal and Torres Strait Islander young people aged 0–24 years, contributing to 22% of all deaths for this age group (Australian Insitute of Health and Welfare, 2024).

At present, there is limited guidance on managing, coping with and rectifying LV in the Australian context for young people, especially as LV is relational to other phenomena such as racism, discrimination and trauma. Some documented strategies to cope with and deter LV include avoidance, such as not identifying as Aboriginal and Torres Strait Islander (Bennett, 2014) or disengaging from family, community, school and Aboriginal and Torres Strait Islander workplaces (Clark, 2017; Webster & Clark, 2024). Such strategies may contribute to further vulnerability and isolation.

### Lateral empowerment

Although young people are the most vulnerable to LV (Svetaz et al., 2020), they are also the future change makers, as exemplified by an Aboriginal community member in Clark (2017).

The future of Aboriginal communities lies with the next generation and therefore a focus on prevention and unity needs to start with young Aboriginal people. (p. 112)

Lateral empowerment (LE) is an emerging term describing interventions that aim to foster autonomy and self-determination for Aboriginal and Torres Strait Islander people. The term lateral love has also been used in the grey literature with messages and a call to action to counteract LV (Butler, 2013). Hence, having power over choices affecting their own lives, LE can be seen as a process to self-determine outcomes that can eliminate and counter LV (Alsop & Heinsohn, 2005). By empowering young people to make their own decisions and choices that counteract or decrease LV, and to support their peers to do the same, young Aboriginal and Torres Strait Islander people can gain a sense of control in their lives (Butler, 2013; Calma & Priday, 2011; Newton, 2016; Priday et al., 2011). Strategies to empower can include arming oneself with knowledge and awareness of LV, propping up support from family, community and workplaces, counselling, positive role modelling and challenging LV where it occurs (Clark et al., 2017). A holistic approach is needed to nurture the culture of Aboriginal and Torres Strait Islander communities and families, and in turn, this will empower families to thrive and improve community and young people’s SEWB (Dudgeon et al., 2023; Durmush et al., 2021; Gee et al., 2014). Empowerment is also gained through “epistemic privilege” from which new and critical knowledge is gained and disseminated by Indigenous populations which counter the epistemic positions of the dominant colonial worldview. The prioritisation of epistemic privilege serves to challenge oppression through the use of Indigenous research methodology and change and challenge colonial social constructs (Vaditya, 2018). This review of grey literature leverages epistemic privilege by empowering the voice and resources of Indigenous populations that do not fall into the colonised academic structure of publication and peer review. Thus, this work amplifies and combines Aboriginal-led community voices into resources (grey literature) that can serve Aboriginal and Torres Strait Islander communities.

### Current objectives

Given that LV and LE are relatively new terms applied to Aboriginal and Torres Strait Islander people, there is limited information and literature in Australia making use of the terms “lateral violence” and “lateral empowerment” among Aboriginal and Torres Strait Islander youth, including a lack of documented experiences and impacts of LV/LE for Aboriginal and Torres Strait Islander youth.

The authors acknowledge that Aboriginal and Torres Strait Islander youth, as it pertains in this review, have been categorised as a homogenous group and have not specifically identified the experience of LV/LE in subcultures of Aboriginal and Torres Strait Islander youth. This scoping review seeks to collate alternative sources of literature, known as “grey literature”, for the presence of LV and LE outside of the published peer-reviewed literature. Grey literature is a field that deals with the production, distribution and access to multiple document types produced at all levels of government, academics, business and organisations in electronic and print formats not controlled by commercial publishing (GreyNet International, 2024). Grey literature provides a platform for Aboriginal and Torres Strait Islander individuals and organisations to publish work and other resources that are not based upon peer-reviewed studies, thereby increasing Aboriginal and Torres Strait Islander expertise in areas which directly impact the community. The methodology examining grey literature leverages epistemic privilege to amplify the knowledge contained in resources and reports collated by Aboriginal and Torres Strait Islander community organisations, and acknowledges that this knowledge is not, and should not be constrained by academic colonised pathways of dissemination. It can provide avenues for voices, resources and accessibility for Aboriginal and Torres Strait Islander people. This grey literature scoping review compliments a scoping review of peer-reviewed literature under review which explored LV and LE in young Indigenous populations across the Canadian, Australian, New Zealand and United States (CANZUS) nations (Hawke et al., 2024). Reviewing grey literature sources will allow further exploration of LV/LE from the perspective of the Aboriginal and Torres Strait Islander community. The purpose of this scoping review is to identify the breadth of grey literature available that pertains to LV and/or LE, and the impacts of LV and LE on young Aboriginal and Torres Strait Islander people; in particular, the aims of this review are to understand what community are saying about LV/LE, gauge understanding of LV/LE within the community and identify any targeted interventions and information relating to LV/LE available to the Aboriginal and Torres Strait Islander community in Australia.

## Materials and methods

### Search strategy

The review of grey literature was conducted following a larger scoping review of peer-reviewed publications (Hawke et al., 2024) which identified several sources of grey literature. While these documents were out of scope for the larger review, the research team acknowledged the value of the grey literature documents for providing insight into the understanding of LV and LE in Aboriginal and Torres Strait Islander communities in Australia. The search was designed to identify all relevant literature and open access documents related to LV and/or LE and Aboriginal and Torres Strait Islander people, and included search engines (Google.com), websites of Aboriginal Community Controlled Organisations (ACCO) and one Indigenous-specific research database (Australian Indigenous HealthInfoNet). Search terms were intentionally broad to return a wide variety of sources and documents. An overview of the hand-searched databases and websites can be found in Table 2.

**Table:** 

Grey Literature search terms	
lateral violence	community violence or bullying
lateral oppression	lateral love
horizontal violence or oppression	lateral empowerment
internalised or internalised oppression/racism/colonialism	lateral healing
peer violence/bullying/abuse	lateral respect
peer racism	lateral forgiveness
intra-racial intra-cultural racism/bullying/violence/abuse	lateral positivity
ingroup racism	lateral kindness
domestic violence	lateral wellbeing
family or familial violence	lateral understanding
family or familial abuse	lateral strength
intergenerational violence or abuse	intra-racial respect/love/healing
infighting	community respect/healing/love/ empowerment/forgiveness
cultural abuse or violence	

The search included government reports, conference proceedings, research reports, fact sheets, health resources, websites and newsletters sourced from a range of organisational websites such as Aboriginal Community Controlled Organisations, government and non-government agencies, research centres, health institutes and non-profit organisations (Table 2).

### Eligibility criteria

The inclusion criteria for this review included any grey literature (as per the Prague definition) published in Australia between January 1980 and September 2023 that specifically reported direct or vicarious experiences of LV and/or LE among youth in Australian Aboriginal and Torres Strait Islander communities. Though the focus of this review is Aboriginal and Torres Strait Islander young people, no age-based exclusion criteria were applied. Documents were excluded if full text was unable to be retrieved. While the search terms were intentionally broad, documents were ultimately excluded if they did not specifically say the words “lateral violence” or discuss themes around lateral empowerment.

### Data extraction

A data extraction form was designed to determine the variables to extract. Two reviewers extracted data (RE, FA) and assessed for consensus. Data items were not limited to specific definitions of violence or empowerment. The data items included but were not limited to 1) synonyms for LV and LE, 2) concepts/triggers that underpin LV and/or LE (e.g., LV: oppression, racism, intergenerational trauma, LE: identity, resilience), 3) behaviours associated with LV or LE (e.g., LV: bullying, covert/overt violence, LE: good mental health, help seeking behaviour) and 4) experiences and impact on health and SEWB outcomes (e.g., family relations, child and adolescent health and well-being, child development and functioning, criminal behaviours, spiritual health and well-being, and connection to culture and identity). Additionally, author background (i.e., whether Aboriginal and/or Torres Strait Islander or non-Aboriginal) was recorded for each document, as were characteristics of the organisation that produced the document (e.g., government body, Aboriginal Community Controlled Organisation, private individual, etc.). Eligible settings included but were not limited to justice systems, government organisations, workplaces, schools, community settings, child protection services, family, homes and health services.

### Project governance and accountability

This project was entirely designed and driven from concept to synthesis by Aboriginal and Torres Strait Islander author specialists (YC, TC, NT, KH) in this field. Non-Aboriginal authors (FA, RE, AB, KP) supported the entire process and aided in screening, extraction and writing of the manuscript. The larger LV project was presented and discussed at an Aboriginal Communities and Families Health Research Alliance (ACRA) forum in November 2021 and since then there has been continual community consultation and yarning about LV and LE with Aboriginal and Torres Strait Islander peoples in SA, facilitated by YC and KH for feedback and perspectives on the project, and preliminary findings.

## Results

### Summary of literature search results

**Figure 1.**
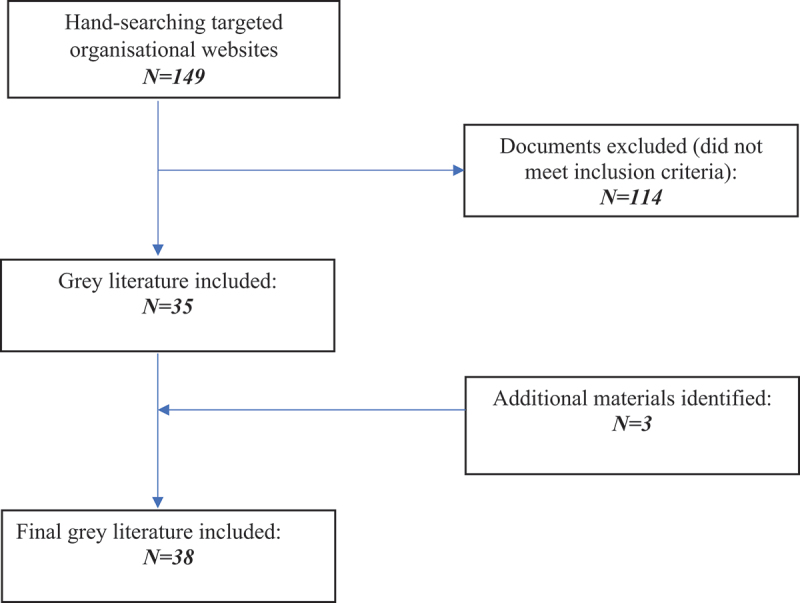
Search and appraisal flowchart.

The grey literature search identified 149 results related to LV and/or LE in Aboriginal and Torres Strait Islander communities. A total of 114 documents were excluded as not meeting the eligibility criteria. During the process of data extraction, a further three documents were identified that also provided insight or resources around LV in Aboriginal and Torres Strait Islander communities, for a total of 38 pieces of grey literature included in this scoping review (see Figure 1). Documents and materials were further stratified into those that predominately focused on themes around LV (21 sources) and those themed around LE (17 sources) (see Table 1).

**Table 1.** Document overview of the included literature.

Document author	Document title	Source type	Concept	Author background	Intervention Type	Themes	Population	Organisation type
**Grey literature with themes around lateral violence (LV)**								
Big River Connections	Aboriginal People and Lateral Violence *(Big River Connections, 2021)*	Program/Workshop	Exploring and addressing LV	Aboriginal/Torres Strait Islander	Workshop exploring and addressing LV	LV	Aboriginal and Torres Strait Islander people	Privately owned Aboriginal and Torres Strait Islander organisation
Boorndawan Willam Aboriginal Healing Service	Men’s resource kit *(Boorndawan Willam Aboriginal Healing Service, 2013b)*	Online resource	Resource kit for men addressing FV and LV	Aboriginal/Torres Strait Islander	Men’s resource kit	FV, LV	Aboriginal and Torres Strait Islander men	ACCO
Boorndawan Willam Aboriginal Healing Service	Women’s resource kit *(Boorndawan Willam Aboriginal Healing Service, 2013c)*	Online resource	Resource kit for women addressing FV and LV	Aboriginal/Torres Strait Islander	Women’s resource kit	FV, LV	Aboriginal and Torres Strait Islander women	ACCO
Boorndawan Willam Aboriginal Healing Service	Children’s resource kit *(Boorndawan Willam Aboriginal Healing Service, 2013a)*	Online resource	Resource kit for children addressing FV and LV	Aboriginal/Torres Strait Islander	Children’s resource kit	FV, LV	Aboriginal and Torres Strait Islander children	ACCO
Australian Human Rights Commission, Aboriginal and Torres Strait Islander Social Justice Commissioner	2011 Social Justice and Native Title Reports – A Community Guide *(Australian Human Rights Commission, 2011a)*	Community guide	Guide summarising two reports which each explore LV in context of impact of colonisation and political agendas	Aboriginal/Torres Strait Islander	N/A	LV	Aboriginal and Torres Strait Islander people	Independent statutory body
Australian Human Rights Commission, Aboriginal and Torres Strait Islander Social Justice Commissioner	Social Justice Report 2011 *(Australian Human Rights Commission, 2011c)*	Report	Annual report related to enjoyment of human rights by Aboriginal people, with focus on LV	Aboriginal/Torres Strait Islander	N/A	LV, human rights-based approach, cultural safety	Aboriginal and Torres Strait Islander people	Independent statutory body
Australian Human Rights Commission, Aboriginal and Torres Strait Islander Social Justice Commissioner	Native Title Report 2011 *(Australian Human Rights Commission, 2011b)*	Report	Annual report that specifically named LV and options for addressing it in relation to native title	Aboriginal/Torres Strait Islander	N/A	LV, colonisation, native title, imbalance of power	Aboriginal and Torres Strait Islander people	Independent statutory body
J. Weir and A. Barnes for Australasian Legal Information Institute (AustLII)	Native Title Newsletter 3/2011, Native Title Conference 2011: Our Country, Our Future *(Weir & Barnes, 2011)*	Newsletter	Summary of the themes coming out of the 2011 Native Title Conference	Aboriginal/Torres Strait Islander	N/A	LV, colonisation	Aboriginal and Torres Strait Islander people	ACCO
Creative Spirits	Bullying and Lateral Violence *(Korff, 2020)*	Online resource	Bullying and LV from a youth perspective	Non-Aboriginal/Torres Strait Islander	N/A	LV, bullying	Aboriginal and Torres Strait Islander people	Private individual
ReachOut Australia	Changing the story: Turning Around Lateral Violence *(ReachOut Australia, & Cox Inall Ridgeway, 2023b)*	Online resource	Information What is LV, Impact of LV on youth, Approaches to help youth experiencing LV	Non-Aboriginal/Torres Strait Islander, in consultation with Aboriginal and Torres Strait Islander people prior to publication	N/A	LV	Aboriginal and Torres Strait Islander youth	Mental health organisation
SafeWork NSW, NSW Government	Lateral Violence *(SafeWork NSW, 2023)*	Online resource	Information about LV	Not stated	N/A	LV	Both Aboriginal and non-Aboriginal people	Government
Child Safety Practice Manual, QLD Government	Domestic and family violence practice kit, “Lateral Violence” *(Queensland Government, 2022)*	Online resource	Information about LV	Not stated	N/A	LV	Aboriginal and Torres Strait Islander children	Government
J. Cedar (LinkedIn blog)	Lateral Violence Is Killing Our Families and Communities *(Cedar, 2023)*	Blog	Blog post discussing impact of being light skinned Indigenous woman and experience of LV	Aboriginal/Torres Strait Islander	N/A	LV, skin colour	Aboriginal and Torres Strait Islander people	Private individual
National Indigenous Times	Lateral Violence is rampant in the Aboriginal community, so what is it? *(Loo, 2022)*	Editorial article	Perspective piece describing LV and the impact and role that political agendas play in LV	Aboriginal/Torres Strait Islander	N/A	LV, government policy as contributing factor	Aboriginal and Torres Strait Islander people	Aboriginal community-led media
Dulwich Centre	Aboriginal narrative practice: Honouring storylines of pride, strength and creativity, Part II: Lateral Violence *(Wingard et al., 2012)*	Book section	Approaches to disrupting lateral violence, including through a theatrical narrative approach to LV	Aboriginal/Torres Strait Islander	Narrative approach	LV	Aboriginal and Torres Strait Islander people	Mental health service
M. Gooda, Aboriginal and Torres Strait Islander Social Justice Commissioner	Eddie Koiki Mabo Lecture: Strengthening Our Relationships Over Land, Territories and Resources: The United Nations Declaration on the Rights of Indigenous Peoples *(Gooda, 2011)*	Lecture transcript	Association between native title and LV	Aboriginal/Torres Strait Islander	N/A	LV, native title, government policy as contributing factor	Aboriginal and Torres Strait Islander people	Private individual
NITV Broadcaster	What is LV and How Do We Deal with Its Many Forms? *(B. Charles, 2023)*	Editorial article	Impact of LV due to colonial policies about Aboriginal identity	Aboriginal/Torres Strait Islander	N/A	LV, skin colour	Aboriginal and Torres Strait Islander people	Aboriginal community-led media
The Daily Telegraph	Aboriginal Wellbeing Conference Focuses on Health and Bullying *(Gulbin, 2015)*	News article	Summary of Aboriginal Wellbeing Conference 2015	Not Stated	N/A	LV, bullying	Aboriginal and Torres Strait Islander people	Media/newspaper
ReachOut Australia	Aboriginal and Torres Strait Islander parents and teenagers *(ReachOut Australia, & Cox Inall Ridgeway, 2023a)*	Online resource	Provides parents with holistic ways to support youth experiencing LV and racism	Aboriginal/Torres Strait Islander	N/A	LV, racism	Aboriginal and Torres Strait Islander parents, youth	Mental health organisation
Australian National Research Organisation for Women’s Safety (ANROWS)	Kunga’s trauma experiences and effects on behaviour in Central Australia *(Bevis et al., 2020)*	Research report	Explores connections between LV and incarceration for Aboriginal women	First author is Aboriginal/Torres Strait Islander with non-Aboriginal/Torres Strait Islander co-authors	Deep listening process	LV, racism, incarceration	Aboriginal and Torres Strait Islander women experiencing incarceration	Research organisation
Education and Health Standing Committee, Western Australia Legislative Assembly	Learnings from the message stick: The report of the Inquiry into Aboriginal youth suicide in remote areas *(Education and Health Standing Committee, 2016)*	Report	Exploring causes of youth suicide	Non-Aboriginal/Torres Strait Islander	N/A	LV, suicide	Aboriginal and Torres Strait Islander youth	Government
**Grey literature with themes around lateral empowerment (LE)**								
Cooperative Research Centre for Aboriginal Health	The Role of Spirituality in Social and Emotional Wellbeing Initiatives: The Family Wellbeing Program at Yarrabah *(McEwan et al., 2008)*	Discussion paper	Explores the role of spirituality in SEWB, considering the outcomes of the Family Wellbeing Empowerment program	Aboriginal/Torres Strait Islander	Family-based SEWB program	SEWB, spirituality	Aboriginal and Torres Strait Islander people	Aboriginal research organisation
R. Carnes, Centre for Rural Regional Law and Justice, Deakin University	Applying a We Al-Li Educaring Framework to Address Histories of Violence with Aboriginal Women *(Carnes, 2016)*	Evaluation report	Evaluation of We Al-li for Kungas course provided by an Aboriginal-owned organisation at Alice Springs Correction Centre	Non-Aboriginal/Torres Strait Islander	Educational course	Patterns of violence, trauma, incarceration	Aboriginal and Torres Strait Islander women experiencing incarceration	University on behalf of privately owned Aboriginal and Torres Strait Islander organisation
Desert Pea Media	Black Lyrical Connection, “Speak 2 Heal” *(Desert Pea Media, 2015)*	Multi-media video	Song writing program	Aboriginal/Torres Strait Islander	Song-writing program	Creative expression, DV, FV	Aboriginal and Torres Strait Islander youth and Elders	ACCO and Aboriginal community-led media
Emerging Minds	Healing through voice, culture, and Country *(Emerging Minds Australia, 2021)*	Multi-media resources, online course	Culturally informed resources for non-Indigenous practitioners working with Aboriginal and Torres Strait Islander families affected by FV and DV	Aboriginal/Torres Strait Islander	N/A	FV, DV	Aboriginal and Torres Strait Islander people	Mental health organisation
Tangentyere Family Violence Prevention Program in collaboration with italk Studios	Old Ways Are Strong: Sharing Knowledge for a Strong Future *(Tangentyere Women’s Family Safety Group and Men’s Behaviour Change Program, 2023)*	Animations	About the strengths of Aboriginal culture and roles of men and women in culture	Aboriginal/Torres Strait Islander	N/A	Creative expression	Aboriginal and Torres Strait Islander people	Media and ACCO
J. Prince for The Healing Foundation	Our Men, Our Healing: Creating Hope, Respect and Reconnection *(The Healing Foundation, 2015)*	Evaluation report	Evaluation of three pilot men’s healing projects	Aboriginal/Torres Strait Islander	Gender-specific, strengths-based approach	Education, employment, health, identity, role modelling, nurturing, resources, safety	Aboriginal and Torres Strait Islander men	ACCO
Inala Wangarra and University of Queensland	Roles and Ritual: The Inala Wangarra Rite of Passage Ball case study *(Poche Centre for Indigenous Health, 2018)*	Multi-media resources	A participatory action research project producing multi-media resources for community and practitioners	Aboriginal/Torres Strait Islander	Creative expression, participatory action	Ritual, coming-of-age, SEWB	Aboriginal and Torres Strait Islander young men	ACCO and university
The Lowitja Institute	Stories of hope and resilience: using new media and storytelling to facilitate “wellness” in Indigenous communities *(The Lowitja Institute, 2010)*	Blog	Project exploring how digital storytelling can facilitate wellness in community	Aboriginal/Torres Strait Islander	Creative expression	SEWB	Aboriginal and Torres Strait Islander people	Aboriginal research organisation
The Healing Foundation and Gawooleng Yawoodeng Aboriginal Corporation	Talking family healing: East Kimberley gathering report *(The Healing Foundation, 2016)*	Report	Report on a gathering of community to facilitate family healing	Not stated	Workshops and Yarning circles	Creative expression, knowledge-sharing, FV, child abuse, inter-generational trauma, suicide	Aboriginal and Torres Strait Islander people	ACCO
The Healing Foundation	Torres Strait and Kaurareg Aboriginal Peoples: Healing Strategy *(The Healing Foundation, 2017)*	Report	Healing practices to promote child safety and increase child wellbeing	Not stated	Strength based cultural practices	Trauma, child and community safety, spiritual healing, self-determination, leadership	Aboriginal and Torres Strait Islander people	ACCO
Edith Cowan University	Valuing Aboriginal and Torres Strait Islander Young Men *(Adams, 2019)*	Videos	Videos exploring how Aboriginal men remain strong and resilient	Aboriginal/Torres Strait Islander	Workshop, strengths-based approach	Creative expression, culture, law, community, leadership	Aboriginal and Torres Strait Islander men	Aboriginal research organisation
A. Jackomos, Victorian Commissioner for Aboriginal Children and Young People	International Human Rights Day Oration: Linking our past with our future: How cultural rights can help shape identity and build resilience in Koori kids *(Jackomos, 2014)*	Lecture transcript	Cultural rights for Koori children to increase SEWB	Aboriginal/Torres Strait Islander	N/A	Strengths-based cultural practices	Aboriginal and Torres Strait Islander children	Private individual
The Healing Foundation	Our Healing Our Way: Alice Springs Healing Forum Report *(Aboriginal and Torres Strait Islander Healing Foundation, 2012)*	Report	LV direct cause of disharmony in community, to counteract need reconnection to country, building cultural strength especially for men	Aboriginal/Torres Strait Islander	Forum	DV, FV, LV, dislocation, suicide, trauma, systemic racism, identity, reconciliation, connection to country, cultural and community strength	Aboriginal and Torres Strait Islander people	ACCO
QLD Centre for Domestic and Family Violence	Strong Women. Hard Yarns. Stories and tips about domestic and family violence *(The Queensland Centre for Domestic and Family Violence Research, 2016)*	Booklet	Violence and what it means for Aboriginal and Torres Strait Islander woman	Aboriginal/Torres Strait Islander	N/A	DV, FV, Elder abuse, lived experience	Aboriginal and Torres Strait Islander women	ACCO
NT Government	The NT Domestic, Family and Sexual Violence Reduction Framework 2018–2028 *(Northern Territory Government, 2018)*	Report	Framework to address violence against women and children in NT	Not stated	N/A	DV, FV, sexual violence, LV	Both Aboriginal and non-Aboriginal people	Government
VIC Government, Dept. of Social Services	Dhelk Dja: Safe our Way – Strong Culture, Strong Peoples, Strong Families *(Australian Government Department of Social Services, 2021)*	Policy	Aboriginal-led agreement committing Aboriginal services, Aboriginal communities and government organisations work collaboratively to end FV	Not stated	N/A	LV, FV, colonisation, dispossession, child removal, discriminatory policies, trauma, disadvantage, racism	Aboriginal and Torres Strait Islander people	Government
The Lowitja Institute for the National Mental Health Commission	Journeys to Healing and Strong Wellbeing Final Report *(The Lowitja Institute, 2018)*	Report	Connection to community to address LV	Aboriginal/Torres Strait Islander	N/A	Trauma-informed care	Aboriginal and Torres Strait Islander people	Aboriginal research organisation

ACCO = Aboriginal Community-Controlled Organisations; LV = Lateral Violence; LE = Lateral Empowerment; SEWB = Social and Emotional Wellbeing; FV = Family Violence; DV = Domestic Violence.

**Table 2.** List of websites hand-searched for grey literature.

Website	URL
Australian Indigenous HealthInfoNet	https://healthinfonet.ecu.edu.au/
Aboriginal Health and Medical Research Council	https://www.ahmrc.org.au/
Lowitja Institute	https://www.lowitja.org.au/
Australian Institute of Aboriginal and Torres Strait Islander Studies	https://aiatsis.gov.au/

Of the 21 documents that addressed LV, the majority (15) were authored by an Aboriginal and/or Torres Strait Islander person with or without non-Aboriginal co-authors. Two documents were authored by a non-Aboriginal person, and another was authored by a non-Aboriginal person who indicated that they consulted with Aboriginal and/or Torres Strait Islander community members. Three documents did not state the cultural background of the author(s). Of the 17 documents that addressed LE, 12 were authored by an Aboriginal and/or Torres Strait Islander person, one was authored by a non-Aboriginal person, and four did not state authors’ cultural background.

### Understanding and experiences of LV

There were several instances of educational documents, materials and resources produced specifically for the Aboriginal and Torres Strait Islander community around LV. Three gender- and age-specific resource kits were freely available from an Aboriginal Community Controlled Organisation (ACCO) (Boorndawan Willam Aboriginal Healing Service, 2013a, 2013b, 2013c). These kits individually targeted men, women and children and defined and described LV, as well as discussing how it can be addressed. One privately owned Aboriginal and Torres Strait Islander organisation ran workshops exploring and addressing the concept of LV within Aboriginal and Torres Strait Islander communities and professional settings (Big River Connections, 2021). A community training and counselling centre published a book describing Aboriginal and Torres Strait Islander narrative practices and previously delivered workshops exploring and addressing the concept of LV within Aboriginal and Torres Strait Islander communities and professional settings (Wingard et al., 2012). One personal blog post detailed lived experience of LV pertaining to racism and Aboriginal and Torres Strait Islander identity (Cedar, 2023). One government resource provided education regarding LV within the workplace and how to create safe workplaces (SafeWork, 2023). Two government resources addressed the impact of LV and family violence on both Aboriginal and Torres Strait Islander children and youth (Education and Health Standing Committee, 2016; Queensland Government, 2022). Two online resources from a mental health service provided information about LV aimed at young people and their parents (ReachOut Australia, & Cox Inall Ridgeway, 2023a, 2023b). In addition, a report from a research organisation outlined the effects of LV in remote communities and the influence of government policies on LV (Bevis et al., 2020). Two reports and a related community guide produced by an independent statutory body discussed LV in the context of native title and human rights (Australian Human Rights Commission, 2011a, 2011b, 2011c).

#### Forms of LV – Internalised racism and Aboriginal and Torres Strait Islander Identity

Different forms of LV identified within the grey literature describing the experiences of Aboriginal and Torres Strait Islander people were discussed within the context of internalised racism whereby Aboriginal and/or Torres Strait Islander youth bully, shame or exclude peers based upon characteristics such as skin colour and bloodline. This brings into question a person’s sense of Aboriginal and/or Torres Strait Islander identity and was identified in two documents, which included a community youth website (ReachOut Australia, & Cox Inall Ridgeway, 2023b) and an editorial article from an Aboriginal and Torres Strait Islander media source (B. Charles, 2023). The authors note internalised racism was also discussed as occurring among Aboriginal and Torres Strait Islander people on the website Creative Spirits (Korff, 2020). However, it is acknowledged by the authors that Indigenous forums (Indigenous & Flynn, 2024), alongside University reports (Sullivan & McLean, 2023) have questioned the authenticity and validity of this website and it was described as perpetuating negative stereotypes of Aboriginal and Torres Strait Islander peoples, due to it being authored and disseminated by a non-Indigenous person, without consultation with Aboriginal and Torres Strait Islander people or community.

#### Causal factors of LV – Government policies and colonisation

Two documents identified causes of LV, which focused upon government political agendas within the National Indigenous Times (Loo, 2022). Additionally, the Social Justice Report reflected how contemporary political factors such as Native Title processes provided a system and environment for LV to thrive within families and communities. Although native title can generate positive changes, it can also fragment communities with opposing viewpoints regarding Native Titles, therefore generating LV to occur (Australian Human Rights Commission, 2011c). A further document discussed colonisation as an underlying cause of LV within the Native Title Newsletter due to policies which require Aboriginal and Torres Strait Islanders to “prove their Aboriginality” (Weir & Barnes, 2011).

### Understanding and experiences of LE

The source of documents addressing LE included four developed within an Aboriginal and Torres Strait Islander controlled research organisation, two from Australian universities, seven from Aboriginal Community Controlled SEWB services, two from state and territory governments, and one from a private individual representing a statutory body. A further one is from a non-Aboriginal mental health and overall health service.

#### Strengthening cultural identity and resilience – lived experience

To strengthen cultural identity and resiliency for Aboriginal and Torres Strait Islander people and address the impact of LV, a diverse set of mechanisms was recommended across the literature. Two documents reported utilising the lived experience of LV among community members, specifically domestic and family violence (DV and FV, respectively) to act as mentors to help support others who have experienced LV (Carnes, 2016; The Queensland Centre for Domestic and Family Violence Research, 2016). Additionally, the power of lived experience of DV was also used to support women by exploring personal narratives of DV and provided anecdotal strategies and approaches for how women have overcome DV to empower other women (The Queensland Centre for Domestic and Family Violence Research, 2016).

#### Family and cultural connection

Intergenerational trauma often underpins LV, and family-based interventions encompassing spiritual connection and healing were shown to improve SEWB, and cultural connectedness within families. This leads to greater capacity for behaviour change, and FV mitigation (McEwan et al., 2008). Demonstrating positive cultural experiences for youth was found to be instrumental in improving the SEWB of Aboriginal and Torres Strait Islander youth experiencing LV, particularly internalised racism. An article on the ReachOut website detailed an information sheet for parents on how best to support their children experiencing LV (ReachOut Australia, & Cox Inall Ridgeway, 2023a). This resource promotes positive cultural experiences, crediting both Aboriginal and Torres Strait Islander role models and celebrating young people’s efforts, talents and successes. Gender-specific Yarning circles were also noted as fundamental to providing a safe space for males and females to discuss their experiences of LV. These spaces lead to the development of culturally informed and appropriate strategies to address LV, empowering community with cultural knowledge and education about men’s and women’s roles, and the importance of positive role models for their families and children (The Healing Foundation, 2015; The Lowitja Institute, 2018).

#### Creative expression of LV experiences

Several grey literature resources used expressive creative modalities to provide education to both Aboriginal and Torres Strait Islander and non-Aboriginal individuals about experiences of LV outside of traditional written forms. In total, five different creative modalities were identified. Multimedia was used by Black Lyrical Connection who developed Speak 2 Heal; a song writing program using local youth, Elders and community members to bring awareness of DV to the community (Desert Pea Media, 2015). Short films titled “Healing through voice, culture and country” narrated by Aboriginal and Torres Strait Islander community members and practitioners were used to educate non-Aboriginal practitioners on how to better support and understand Aboriginal and Torres Strait Islander youth and people experiencing DV and FV (Emerging Minds Australia, 2021). Cultural education for Aboriginal and Torres Strait Islander men and women about the strength of Aboriginal and Torres Strait Islander culture and the traditional gender roles used as protective factors against DV and FV was expressed and presented in animation form “Old Ways are Strong; Sharing Knowledge for A Strong Future” (Tangentyere Women’s Family Safety Group and Men’s Behaviour Change Program, 2023). Digital storytelling “Stories of hope and resilience” was used as a way for Aboriginal and Torres Strait Islander youth and Elders to have a space to share their narratives and cultural knowledge as a mechanism to improve SEWB (The Lowitja Institute, 2010). Digital storytelling and film were also utilised as a modality for young Aboriginal and Torres Strait Islander men to share their cultural stories and strategies to remain strong and resilient in the face of adversity (Poche Centre for Indigenous Health, 2018).

## Discussion

This overview of grey literature exploring LV and LE of Aboriginal and Torres Strait Islander youth enriches the understanding of what Aboriginal and Torres Strait Islander communities are saying about LV/LE, what communities understand of LV/LE and if there are any targeted interventions or information relating to LV/LE. Most of the grey literature available was produced by Aboriginal and Torres Strait Islander-controlled organisations and businesses and is written by community members. This demonstrated the integral role that communities have in creating resources that are led by Aboriginal and Torres Strait Islander people for Aboriginal and Torres Strait Islander people. This also highlights that Aboriginal and Torres Strait Islander communities are aware of LV/LE and are proactive about addressing it. A breadth of literature concerning the different creative modalities for addressing LV/LE not only demonstrates the adaptive nature of support available but also the difference in needs and diversity of community settings.

Forms of LV identified within the grey literature detailing the experiences of Aboriginal and Torres Strait Islander youth were largely discussed within the context of internalised racism which were disseminated via youth-based website Reach Out. The lived experience of LV by way of “questioned Aboriginality” and Aboriginal authenticity due to skin colour was also shared via personal blogs disseminated via Aboriginal and Torres Strait Islander Broadcaster NITV. Such questioning is believed to stem from colonisation and historical government policies that at first categorised Aboriginal and Torres Strait Islander people into colours and blood quantum such as “quadroons”, “quarter and half-castes” and “full bloods” (National Inquiry into the Separation of Aboriginal and Torres Strait Islander Children from their Families, 1997). Ironically Aboriginal and Torres Strait Islander people are still required to “prove their Aboriginality”, to governments and in addition to each other, causing fragmentation within communities contributing to violence (B. Charles, 2023; Gooda, 2011). The firsthand recounts of intercommunity LV demonstrate that in-group racism is an ongoing concern, and that there is a desire from the community to raise awareness of its presence through experiences and story. This was especially poignant for Aboriginal and Torres Strait Islander youth.

The grey literature provided a breadth of historical truth-telling by way of greater critical discussion regarding causes related to colonisation and the political agendas of successive Australia (Bevis et al., 2020; Weir & Barnes, 2011). A report developed by the Social Justice Commissioner described how contemporary political factors such as Native Title create an environment for LV to thrive within families and communities (Australian Human Rights Commission, 2011b). By provoking friction within communities relating to discussion such as identity, mining proposals and Native title, parallel to government disregard, or dictation of community hierarchy and voices, oppression caused by political agendas encourages LV (Loo, 2022).

The grey literature demonstrates that Aboriginal and Torres Strait Islander communities are taking an active role in addressing LV within their communities and providing resources and strategies to address and identify LV. Yet there remained a paucity of resources and interventions available for Aboriginal and Torres Strait Islander youth, with only three organisations disseminating youth-specific resources addressing the impacts of LV on youth, forms of LV and strategies for adults on how to support youth experiencing LV (Big River Connections, 2021; Queensland Government, 2022; ReachOut Australia, & Cox Inall Ridgeway, 2023a, 2023b). Further youth targeted resources and interventions directly targeting experiences of in-group racism are needed to address LV among this cohort.

LE was highlighted within the literature as a mechanism to build capacity for healing, learning and adaption to both counteract and address the impact of LV on Aboriginal and Torres Strait Islander people by strengthening cultural identity and resiliency. Strength-based approaches focused on family-based approaches demonstrated positive cultural practices to strengthen cultural connectedness for youth and adults alike. These strategies were identified as key to increasing SEWB and, in turn, may serve to decrease incidences of LV. Furthermore, the grey literature highlighted the strength of gender-based strategies and support groups to strengthen cultural identity and improve SEWB among Aboriginal and Torres Strait Islander peoples. Cultural education for Aboriginal and Torres Strait Islander men and women about the strength of their culture and the traditional gender roles used as protective factors against DV and FV was expressed and presented in animation form ‘Old Ways are Strong: Sharing Knowledge for A Strong Future (Tangentyere Women’s Family Safety Group and Men’s Behaviour Change Program, 2023).

The use of creative modalities for the expression of LV/LE reflects its utility, particularly in providing different options and opportunities for expression and healing among Aboriginal and Torres Strait Islander communities, and allowing for the dispersion of information to a wider audience that transcends age and gender. More importantly, this ensures the continued sharing of knowledge between youth and Elders to strengthen cultural identity and strength as demonstrated by “Stories of hope and resilience” (The Lowitja Institute, 2010) creating a space for Aboriginal and Torres Strait Islander youth and Elders to share their narratives and cultural knowledge as a mechanism to improve SEWB. The sharing of cultural information was also demonstrated as a mechanism to teach men and women of the traditional roles in community which have been negatively impacted by colonisation as a way to enable cultural teachings and learnings to be carried down to Aboriginal and Torres Strait Islander young people and youth to create change to empower and reduce LV (Tangentyere Women’s Family Safety Group and Men’s Behaviour Change Program, 2023).

### Strengths and limitations

The comprehensive grey literature review is a noteworthy strength that enabled the voice of both Aboriginal and Torres Strait Islander people pertaining to LV/LE to be highlighted, who otherwise are often omitted due to being outside of the scope of published peer reviewed articles. It must be acknowledged that there are few traditional search strategies and methods for obtaining all grey literature, and while extensive hand searching was undertaken, grey literature may be missed due to the nature of fragmented and targeted publications. The authors’ views of where to search for grey literature concerning LV may have introduced bias to the results. This review was limited by the types of grey literature sources as Western methods bias written online literature. This means that sources such as podcasts, TV series and informal gathering/Yarning support groups were not included if they were not associated with written resources online. In future, it would be pertinent for future grey literature searches to include these forums. A further limitation concerns the lack of stratification or examination of subgroups within the focus population (e.g., LGBTQIA+ people, homeless people, single-parent families and adolescents or children in out-of-home care). It is acknowledged that LV may impact some groups disproportionately within our focus population, and future reviews should independently seek to examine literature associated with vulnerable populations.

This review drew on strengths by reviewing grey literature, which is more likely than commercial research publications to feature the voices and experiences of Aboriginal and Torres Strait Islander people who have traditionally been excluded from or underrepresented in commercial or peer-reviewed literature. However, the review was limited by an overall lack of materials relating to LV/LE, which are emerging topics within the Aboriginal and Torres Strait Islander community.

## Conclusion

Findings of the grey literature review revealed that Aboriginal and Torres Strait Islander communities within Australia are working to raise awareness of LV/LE and are taking active steps to address and counteract the impacts on their communities. The review elucidated that Aboriginal and Torres Strait Islander youth are exposed to in-group racism, family violence and bullying/harassment. Although the review noted few resources available to support youth in coping with LV and harnessing LE, there is a clear need for targeted interventions. Approaches found to be the most frequently offered utilise the creative expression of LV and incorporate strength-based interventions. This method of support is proposed to aid in integrating positive cultural experiences with gender-specific cultural traditions. Supports were noted to include family strategies occasionally to create a wholistic generational impact, reducing the ongoing occurrence of LV. Unfortunately, the sparsity of grey literature reinforces the notion that there still exists a need for resources targeted to whole family healing from the impact of LV.

## Data Availability

The authors confirm that the data supporting the findings are found within the article. Any additional data can be made available upon reasonable request to the first author.
